# Assessing the methanogenic activity of microbial communities enriched from a depleted reservoir

**DOI:** 10.1093/femsec/fiaf040

**Published:** 2025-04-15

**Authors:** Arianna Vizzarro, Annalisa Abdel Azim, Ilaria Bassani, Ruggero Bellini, Nicolò Santi Vasile, Candido Fabrizio Pirri, Francesca Verga, Barbara Menin

**Affiliations:** Centre for Sustainable Future Technologies, Fondazione Istituto Italiano di Tecnologia, Via Livorno 60, 10144 Turin, Italy; Department of Environment, Land and Infrastructure Engineering, Politecnico di Torino, Corso Duca degli Abruzzi 24, 10129 Turin, Italy; Centre for Sustainable Future Technologies, Fondazione Istituto Italiano di Tecnologia, Via Livorno 60, 10144 Turin, Italy; Department of Environment, Land and Infrastructure Engineering, Politecnico di Torino, Corso Duca degli Abruzzi 24, 10129 Turin, Italy; Department of Applied Science and Technology, Politecnico di Torino, Corso Duca degli Abruzzi 24, 10129 Turin, Italy; Centre for Sustainable Future Technologies, Fondazione Istituto Italiano di Tecnologia, Via Livorno 60, 10144 Turin, Italy; Centre for Sustainable Future Technologies, Fondazione Istituto Italiano di Tecnologia, Via Livorno 60, 10144 Turin, Italy; Centre for Sustainable Future Technologies, Fondazione Istituto Italiano di Tecnologia, Via Livorno 60, 10144 Turin, Italy; Department of Environment, Land and Infrastructure Engineering, Politecnico di Torino, Corso Duca degli Abruzzi 24, 10129 Turin, Italy; Centre for Sustainable Future Technologies, Fondazione Istituto Italiano di Tecnologia, Via Livorno 60, 10144 Turin, Italy; Department of Applied Science and Technology, Politecnico di Torino, Corso Duca degli Abruzzi 24, 10129 Turin, Italy; Department of Environment, Land and Infrastructure Engineering, Politecnico di Torino, Corso Duca degli Abruzzi 24, 10129 Turin, Italy; Centre for Sustainable Future Technologies, Fondazione Istituto Italiano di Tecnologia, Via Livorno 60, 10144 Turin, Italy; Istituto di Biologia e Biotecnologia Agraria, Consiglio Nazionale delle Ricerche, Via Alfonso Corti 12, 20133 Milan, Italy

**Keywords:** hydrogenotrophic methanogenesis, underground biomethanation reactor (UMR), hydrogen, enrichment culture, carbon source

## Abstract

Using a depleted gas reservoir as a natural reactor is a novel approach for microbial methanation of hydrogen (H_2_) and carbon dioxide (CO_2_) into methane (CH_4_). This approach, known as underground biomethanation reactor (UMR), could enable the simultaneous valorization of geologically sequestered CO_2_ and the excess renewable energy, stored in the form of H_2_ in the same formation as the CO_2_. In this study, we explore the possibility to trigger biomethanation from formation water sample by testing various carbon sources (CO_2_, trypticase peptone, glucose, and acetate) in batch test with a defined mineral medium. Obtained results show that trypticase peptone supplementation greatly increased methane production and the enrichment of methanogenic archaea, outperforming alternative carbon sources. 16S rRNA amplicon sequencing of the enriched consortia revealed that supplementation of trypticase peptone and a mixture of H_2_:CO_2_ (80:20), resulted in the selection of a mixed culture dominated by microorganisms assigned to the *Methanothermobacterium, Garciella*, and *Caminicella* genera. Furthermore, KEGG (Kyoto Encyclopedia of Genes and Genomes) and COG (Clusters of Orthologous Genes) predictive functional analyses underline a possible syntrophic relationship, enhancing the conversion of H_2_ and CO_2_ into CH_4_. This work lays the groundwork for biologically exploiting a depleted gas reservoir by implementing the UMR technology.

## Teaser

Investigating methanogenic potentials of the microbiota native to a depleted natural gas reservoir.

## Introduction

In September 2020, the European Commission urged that carbon dioxide (CO_2_) and greenhouse gase emissions be further reduced to 55% and that the percentage of renewable energy be increased from 20% to 32% by 2030. Long-term goals to become carbon-neutral by 2050 are also being reviewed (Lagioia et al. 2023). Developing and integrating renewable energy sources and reducing energy waste are pivotal to improving energy production and utilization. Furthermore, it is critical to design a system with a large capacity for long-term storage due to the seasonal fluctuation of renewable energy sources. To fulfill this purpose, hydrogen (H_2_), which is currently mainly produced from excess renewable electricity through electrolysis, provides a useful renewable energy carrier that could be stored in underground geological formations (Panfilov et al. 2016, Thiyagarajan et al. 2022, Okere et al. 2024). UHS is evolving as a critical component in the transition to a hydrogen-based energy system. Underground hydrogen storage (UHS) leverages geological formations such as depleted reservoirs, aquifers, and salt caverns to store hydrogen, addressing the challenges of renewable energy variability ( Taiwo et al. 2024). Understanding hydrogen's interaction with geological material and the interplay between microbial and geochemical reactions is essential for ensuring safe and efficient storage (Okere et al. 2024). In fact, reservoir mineralogy and brine composition can significantly affect microbial activity and hydrogen storage performance (Bassani et al. 2023, Vasile et al. 2024).

In the last decades, early research on hydrogen storage showed unexpected behavior in reservoirs where H_2_ was injected, namely a decrease in pressure was observed due to the reduction of H_2_ and CO_2_ into CH_4_ (Panfilov et al. 2010, 2016, RAG 2020, Thaysen et al. 2021, Tyne et al. 2021, Hellerschmied et al. 2024). In-depth microbiological studies of these sites revealed that methane production was mainly due to the activation of methanogenic microorganisms (Šmigáň et al. 1990). Using these depleted reservoirs as natural reactors for the microbial methanation of H_2_ and CO_2_ is an innovative approach, called underground biomethanation reactor (UMR), proposed by Mikhail Panfilov (Panfilov et al. 2010). UMR consists in injecting H_2_ and CO_2_ from various sources into underground gas storages during energy production peaks. The injected gases are converted into a methane-rich gas mixture, which is withdrawn during high energy demand peak, helps to balance the grid by ensuring a continuous and reliable supply of electricity (Bellini et al. 2022). UMR serves a dual purpose: the valorization of CO_2_ by converting it into CH_4_ and the storage of surplus energy generated by renewable sources in the form of chemical energy (Benetatos et al. 2021). Moreover, this transformation enhances the energy potential of the stored gas, as CH_4_ has a higher energy content (36 MJ/m^3^) compared to H_2_ (10.88 MJ/m^3^) (Bellini et al. 2022). In the case of underground bio-methanation, methanogenic archaea act as biocatalysts, triggered by the injection of CO_2_ and H_2_ which are used as carbon and electron sources for methanogenic metabolism (R1).


\begin{eqnarray*}
4\ {{\mathrm{H}}}_2 + {\mathrm{C}}{{\mathrm{O}}}_2 \to {\mathrm{C}}{{\mathrm{H}}}_4 + 2\ {{\mathrm{H}}}_2{\mathrm{O}}\,\,\,\,\,\,\,\,\,\,\,\, -135.6\ \Delta {{\mathrm{G}}}_{\mathrm{o}}\rm {'}\ \mathrm{ kJ}/\mathrm{ reaction}\left( {{\mathrm{R}}1} \right).
\end{eqnarray*}

A subsurface porous medium, like a depleted gas reservoir, might function similarly to membrane, trickled-bed or fixed-bed reactors that are currently used for *in situ* and *ex situ* biomethanation processes, due to the high surface-to-volume ratio. Compared to reactors, underground structures provide naturally porous environments with a large pore volume and flow capacity (Strobel et al. 2020). Moreover, a critical point is the presence of suitable living conditions for hydrogenotrophic methanogens (HM) inside the reservoir (Bellini et al. 2024) and it is important to consider that other microorganisms, such as homoacetogenic bacteria (R2) and sulfate-reducing bacteria (SRB, R3) inhabit subsurface environments, competing with methanogenic archaea for the supply of H_2_ (Ranchou-Peyruse et al. 2019, Dopffel et al. 2021, Bassani et al. 2023)


\begin{eqnarray*}
4\ {{\mathrm{H}}}_2 + 2{\mathrm{ HCO}}_3^ - + {{\mathrm{H}}}^ + \to {\mathrm{Acetat}}{{\mathrm{e}}}^- + 4\ {{\mathrm{H}}}_2{\mathrm{O}}\,\,\,\,\,\,\,\, -104.6\,\mathrm{ kJ}/\mathrm{ reaction}\ \\\left( {{\mathrm{R}}2} \right),
\end{eqnarray*}

\begin{eqnarray*}
4\,{{\mathrm{H}}}_2 + {\mathrm{SO}}_4^{2 - } + {\mathrm{H}} + \to {\mathrm{H}}{{\mathrm{S}}}^- + 4\ {{\mathrm{H}}}_2{\mathrm{O}}\,\,\,\,\,\,\,\,\,\,\,\,\,\, -151.9\ \mathrm{ kJ}/\mathrm{ reaction}\ \left( {{\mathrm{R}}3} \right).
\end{eqnarray*}

Key factors influencing microbial proliferation include temperature, salinity, and pH. Thaysen et al. (2023) categorized reservoirs into four levels of microbial activity risk based on these parameters. For example, reservoir, whose temperature is below 55°C, might be exposed to high microbiological risk, providing potential conditions for HM activation (Thaysen et al. 2023) even though assays coupling batch cultivation and bio-geochemical simulation did not reveal potential methanogenic activity due to low amount of cell number and limited nutrient availability (Bellini et al. 2024, Vasile et al. 2024). In fact, although batch assays do not provide an exhaustive insight into natural microbial metabolic capacities, when combined with biogeochemical simulation studies, they become a useful tool for microbiological risk assessment.

Field studies of UMR technology demonstrate its potential for in situ biomethanation in depleted hydrocarbon reservoirs (DHRs) and deep aquifers, yet challenges remain. Hellerschmied et al. (2024) reported an 84.3% hydrogen recovery rate after 285 days in a DHR, with microbial activity influencing gas composition and migration. Similarly, Ranchou-Peyruse et al. (2024) identified HM in deep aquifers, but competition with SRB and homoacetogens affected efficiency. Additional challenges included H_2_ dissolution in brine, potential interactions with reservoir rock, and fluctuating redox conditions that impacted microbial activity. These findings highlight the need for precise site selection and monitoring while demonstrating the value of integrating experimental assays with biogeochemical models. Additional challenges include H_2_ migration into cushion gas, dissolution in brine, and potential reservoir rock interactions, though no significant mineral dissolution was detected (Hellerschmied et al. 2024). Furthermore, fluctuating redox conditions and variable sulfate concentrations impacted methanogenic activity in deep aquifers, emphasizing the need for precise site selection and monitoring strategies (Ranchou-Peyruse et al. 2024). These findings underscore the feasibility of UMR technology while highlighting key constraints that must be addressed for large-scale implementation.

To fully unlock the potential of UMR deeper insights into the chemical and biological processes within these systems are essential (Dopffel et al. 2021, Bassani et al. 2023). Microbial populations specifically selected or evolved methanogenic strains, capable of competing for H_2_ as an energy source for the reduction of CO_2_ to CH_4_ could also be artificially introduced within suitable geological structure to implement the UMR concept (Bellini et al. 2022). In the present study, we investigated a new enrichment strategy and the use of different carbon sources (i.e. CO_2_, trypticase peptone, glucose, and acetate) for specifically stimulating hydrogenotrophic methanogenesis and enriching the methanogenic consortium populating reservoir formation fluids. Upon preliminary screening, analyses based on the sequencing of V3–V4 regions of 16S rRNA bacterial and archaeal genes were carried out on the batches supplied with the carbon source that induced higher methanogenic activity. Eventually, the Kyoto Encyclopedia of Genes and Genomes (KEGG) and the Clusters of Orthologous Genes (COG) predictive functional annotations were used to highlight the metabolic pathways potentially involved in the process. This work lays the groundwork for the biological exploitation of the studied depleted gas reservoir by the stimulation of indigenous methanogenic archaea, implementing UMR technology.

## Materials and methods

### Sample collection and hydro-chemical analyses

The inoculum utilized in this study was obtained from formation water from a reservoir located in the center of Italy at a depth of 1065 m with an initial pressure of approximately 150 bar and a temperature of about 48°C. The geological formation consisted of sandstone with a porosity comprised 20–25%, and a permeability of few millidarcy. Formation waters were sampled using the BAILER system, transport, store and analyzed as described in detail by Bassani et al. (2023). Following collection, water samples were sent to the laboratory and kept in a dark chamber at 4°C. Analyses did not include fluid samples collected from the first descent. Within 24 h of sampling, the remaining formation water was processed for further microbiological characterization, as detailed below, while an aliquot was promptly taken for hydrochemical investigation (Table S1).

The microbial biomass utilized as the inoculum was collected by filtering 300 ml of formation water using a vacuum suction via Millipore Express PLUS PES filtering membranes (0.22 µm; Merck Millipore, Burlington, MA, USA). The optimal volume of formation water to be filtered and used as the inoculum was selected based on preliminary quantive PCR (qPCR) tests, carried out on three different volumes of formation water (200, 300, and 500 ml) resulting in the optimal volume for experiment operation (data not shown). Filtration and inoculation were carried out in a vinyl anaerobic chamber (Coy Laboratory Product, Grass Lake, USA) which is constantly supplied with H_2_, CO_2_, and N_2_. Batch cultures were carried out in 158 ml sterile serum flasks. Each serum flask was inoculated with one filter (0.22 µm; Merck Millipore, Burlington, MA, USA) on which the biomass was collected, and provided with 32 ml of nutrient medium. The headspace was filled with 2 bar of a H_2_:CO_2_ blend in a 4:1 ratio (V/V).

### Mineral medium and batch cultures

The mineral media composition was defined by considering the hydro-chemical characteristics of the formation water (Table S1) and to favor hydrogenotrophic methanogenesis over other hydrogenotrophic metabolisms, such as homoacetogenesis and sulfate reduction. The mineral medium was prepared according to Rodrigues-Oliveira et al. (2023) with slight modifications. In particular, the pH was adjusted to 7 with HCl and the medium was prepared without the addition of reducing agents such as resazurin or cysteine-HCl, to facilitate the assessment of the natural reduction potential of the microbial community (Szafranek-Nakonieczna et al. 2019). The tightness of the bottles was monitored through micro-GC (fusion 2-module Inficon, Bad Ragaz, Switzerland). Moreover, a Trace Element (TE) solution was added to fulfill the requirements for methanogenesis (Rodrigues-Oliveira et al. 2023). The batches were prepared in triplicate with either 1 g/l trypticase-peptone, 1 mM glucose, and 1 mM acetate, respectively. Control samples were prepared with inoculum and mineral medium, without the addition of any organic carbon source (Imachi et al. 2020). For each experimental condition, an inoculum-free medium batch was prepared as the negative control. All batches were supplemented with H_2_ and CO_2_ (80:20) through sterile filters (MILLEX 0.22 µm, Merck KGaA, Darmstadt, Germany) by using a gas manifold (Tecnodelta, Italia) equipped with single gas mass flow controllers (Burket, Germany). Batches were incubated at 2 bar and 48°C horizontally to improve gas–liquid mass transfer in an orbital shaker incubator (Biosan orbital agitator-incubator ES-20/60, Riga, Latvia) at 90 r/m for 30 days.

After 30 days, the culture displaying the highest methanation yield was further propagated by transferring 10 ml of cell suspension to a fresh mineral medium followed by 1-month incubation under the same conditions. Every 10 days, after liquid sampling, the amount of liquid taken from the batch was replaced by a fresh mineral medium to provide new nutrients and maintain constant gas/liquid ratios.

### Analytical methods

#### Pressure measurements and gas chromatography analysis

Headspace gas measurements (LEO1 digital manometers KELLER, Winterthur Switzerland) were taken once a week and at the end of the 30 days enrichment phase. Headspace pressure of cultures obtained by trypticase peptone treatment was measured every 3 days during 30 days of incubation. When a pressure drop was detected, the gas composition in the serum flasks was determined using gas chromatography with a Micro GC Fusion system (Inficon, Bad Ragaz, Switzerland). Gas samples were collected using a 5 µl Gastight Syringe (Hamilton 1700 Series Syringes, Bonaduz, Switzerland) and directly fed into the Micro GC. Gas concentrations were determined for H_2_, O_2_, N_2_, CH_4_, CO_2_, and H_2_S. These data allow to estimate the CH_4_ production according to the ideal gas law. After each measurement, the batches were supplemented with 2 bar of H_2_ and CO_2_.

Statistical significance (*P*-value < .05) of gas consumption was assessed using one-way ANOVA and Turkey's multiple comparisons test, with a single pooled variance.

#### Volatile fatty acids analysis

For each experimental condition, short-chain volatile fatty acids (scVFA) were measured once a week. Liquid samples were centrifuged for 10 min at 10 000 r/m in a MicroCL 17R Centrifuge (Thermo Fisher Scientific, Waltham, MA, USA) and the supernatant was filtered through 0.22 µm filters (Scharlab, Barcelona, Spain). The high-pressure liquid chromatography method developed by Bellini et al. (2024) for detecting acetic, propionic, formic, lactic, *n*-butyric/iso-butyric, and *n*-valeric/iso-valeric acid was applied.

### DNA extraction and quantitative PCR analysis

The DNA was extracted from inoculum formation fluids (T_0_) and each experimental condition at time 7, 14, 20, and 30 days with DNAeasy Power-Soil Pro Kit protocol (Qiagen, Hilden, Germany) following the modified protocol as detailed in Bassani et al. (2023). The quality and quantity of the extracted DNA were assessed using a Genova Nano micro-volume spectrophotometer (Jenway, Cole Parmer Inc) and Qubit fluorimeter (Life Technologies, Carlsbad, CA, USA).

The copy number of the *mcrA, dsrB*, and *fhs* genes targeting HM, AB, and SRB, respectively, was determined by qPCR, as reported by Bellini et al. (2024). *mcrA* is frequently utilized as a molecular tool for enumerating HM archaea. The *mcrA* gene encodes the alpha subunit of methyl coenzyme M reductase, an enzyme involved in the final step of methane production (Hales et al. 1996, Nyyssönen et al. 2012). The *dsrB* gene is commonly targeted for the enumeration of SRB; this gene encodes the beta subunit of dissimilatory sulfite reductase, an enzyme involved in sulfate reduction (Foti et al. 2007). The *fhs* gene is associated with AB. This gene encodes the formyltetrahydrofolate synthetase enzyme, which is involved in the Wood–Ljungdahl pathway, a key pathway utilized by acetogenic bacteria for acetate synthesis (Leaphart and Lovell 2001, Leaphart et al. 2003, Xu et al. 2009). Primer sequences utilized are reported in Table S2. The approach employed yielded the lowest amplicon concentrations: 1.33 × 10^2^, 1.85 × 10^2^, and 1.76 × 10^2^ copy/ml for *mcrA, dsrB*, and *fhs*, respectively. As a result, samples exhibiting a number of copies below these cutoff points are labelled as “not determined” (n.d.).

Statistical significance (*P*-value < .05) of *mcrA* copies/ml variation was assessed using one-way ANOVA and Turkey's multiple comparisons test, with a single pooled variance.

### Assessment of microbial community composition

The DNA samples extracted from inoculum formation water (T_0_), trypticase peptone enriched culture (TP) at T_30_ and T_60_ and the first subculture at T_30_ (T_30_TP.1) were analyzed through next generation sequencing (NGS). The 16S amplicon sequencing targeted the V3–V4 region of bacterial and archaeal 16S rRNA was carried out by use respectively 357wF-785R and Arch340wF-Arch806R pairs of primer (Table S2). Libraries were prepared according to Illumina's 16S metagenomic sequencing library preparation protocol. Sequencing of the V3–V4 hypervariable region was conducted using Illumina MiSeq (Element Biosciences, San Diego, CA) with a 300 bp paired-end module by IGA Technology Service, according to the QIIME pipelines (Caporaso et al. 2010), using the USEARCH algorithm (version 8.1.1756, 32-bit), as described by Bassani et al. (2023).

After sequencing, reads were demultiplexed, merged into consensus pseudo-reads, and trimmed to remove low-quality bases, discarding those under 200 bp. Microbiome analysis was conducted using QIIME2 (Caporaso et al. 2010), with operational taxonomic units (OTUs) clustered at 97% similarity via the USEARCH algorithm. Taxonomic classification was performed using the RDP classifier and the 16S GreenGene database, with a confidence threshold of 0.50 and reference database (Silva 138) (Quast et al. 2013, Yilmaz et al. 2014). The Alpha diversity was estimated using the Shannon diversity index, whereas the beta diversity was estimated with the Bray–Curtis dissimilarity statistic index and the distant matrix obtained was graphically represented by principal component analysis (PCoA) and displayed by the Emperor tool (https://biocore.github.io/emperor/) (Bassani et al. 2023). The graphs representing the relative abundance of most abundant OTUs were obtained using Graph pad Prism 10.1.2. The data for this study have been deposited in the European Nucloeotide Archive (ENA) at EMBL-EBI under accession number PRJEB76268.

### Predictive functional analysis of metabolic pathways in dominant genera

A predictive functional analysis was conducted to characterize KEGG and COG functional categories in the most abundant genera (≥1% relative abundance) (Kanehisa and Goto 2000, Galperin et al. 2021). The analysis, performed by IGA Technology Services (Udine, Italy) as described by Bassani et al. (2023), determined the average number of genes assigned to each KEGG and COG category per genome. To predict the metabolic functions of dominant bacterial and archaeal genera, genomic data were retrieved from the IMG/M database (Chen et al. 2023). Genomes without available data were excluded, and bacteria and archaea were analyzed separately. KEGG Pathway and Module analyses mapped KEGG ortholog (KO) annotations to pathway and module IDs using the KEGGREST Bioconductor R package. KO terms were extracted for each genus, translated into pathway/module IDs, and aggregated by taxonomic genus (KEGG releases 105.0+/12–28, December 22, and 105.0+/02–06, February 23). KO annotations without pathway assignments were separately aggregated. COG analysis mapped gene annotations to functional categories using COG database sFTP release 2020. Genes were assigned to COG categories, with aggregated statistics calculated per genus. Unclassified genes were negligible.

## Results

### Screening of batch cultures with different carbon sources

#### Gas measurements and gas chromatography analysis

**Figure 1.**
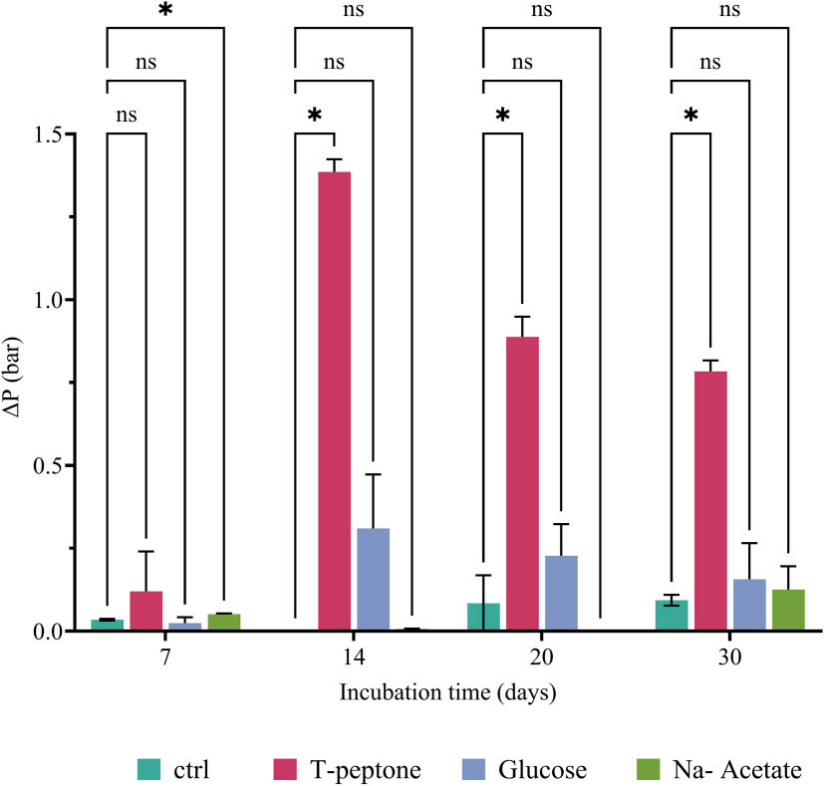
Gas consumption during the incubation time of batch test with different carbon sources. Gas consumption is expressed as ∆*P*. The error bars show the standard deviation (*n* = 3). Statistical significance (*P*-value < .05) between treated and ctrl conditions is shown in the figure, while *P*-values are displayed in Table S3.

Variations in headspace partial pressure were used to assess the microbial activity. The effect of different carbon sources on gas consumption, which was determined from the partial pressure variation between two successive measurements, is illustrated in Fig. 1. Throughout the incubation period, no significant gas consumption was observed when no organic carbon was provided and a negligible CH_4_ yield was achieved at the end of the experiment (0.005 g CH_4_/g CO_2_). Conversely, in the case of trypticase peptone, significant gas consumption was observed from day 14 and continued throughout the experiment. This condition exhibited the highest CH_4_ yield (0.19 g CH_4_/g CO_2_). Gas consumption with glucose was detected in relatively constant amounts from day 14, but at lower levels compared to the trypticase peptone condition. The total yield of CH_4_ yield was also lower than that of trypticase peptone (0.08 g CH_4_/g CO_2_). In the presence of Na-Acetate, no gas consumption was detected until day 30, and the CH_4_ yield was the lowest among the tested conditions (0.014 g CH_4_/g CO_2_).

#### Volatile fatty acids analysis

**Figure 2.**
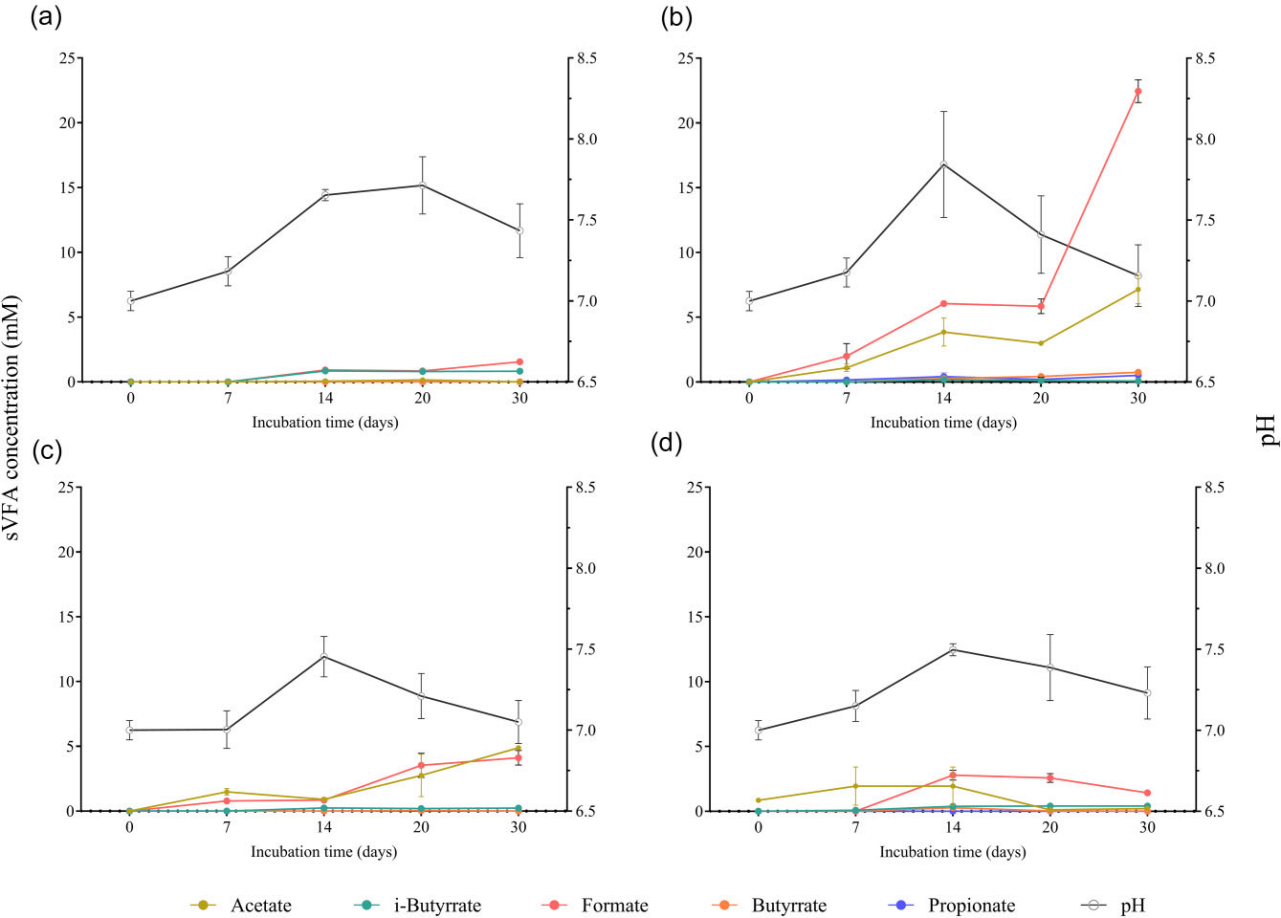
Variation of VFAs concentration and pH during the enrichment phase of batch test with different carbon sources: ctrl condition (a), trypticase peptone (b) glucose (c), and Na-acetate (d). Variation of VFAs concentration is expressed in mM during the enrichment phase. The error bars show the standard deviation (*n* = 3).

During the entire incubation period, across all conditions, higher concentrations of acetate and formate were observed compared to propionic and *n*-butyric acid whose concentrations did not exceed 0.50 and 0.75 mM, respectively (Fig. 2). The control condition, not supplemented with any organic carbon source, showed the lowest amount of all volatile fatty acids (VFAs) analyzed (Fig. 2a). In presence of trypticase peptone (Fig. 2b), remarkably higher levels of formate were produced compared to any other tested condition. The concentration of formate increased over time, with a notable rise between day 20 and day 30 (up to 22.5 mM), while not overcoming 4 mM in the other conditions. Similarly, a stepwise increase in acetate levels was observed in trypticase peptone-supplemented batches (up to 7.14 mM). In the condition supplemented with glucose (Fig. 2c), the concentration of acetate increased steadily over time, reaching 4.88 mM. This suggests that acetate production was sustained in the presence of trypticase peptone and glucose. Eventually, in the Na-acetate supplemented test, acetate production or accumulation was observed until day 14, being almost completely consumed by the end of the experiment, suggesting that either acetate was being utilized by microorganisms or undergoing other forms of metabolic transformation in the system (Fig. 2d).

#### Assessment of microbial community composition with different carbon sources

**Figure 3.**
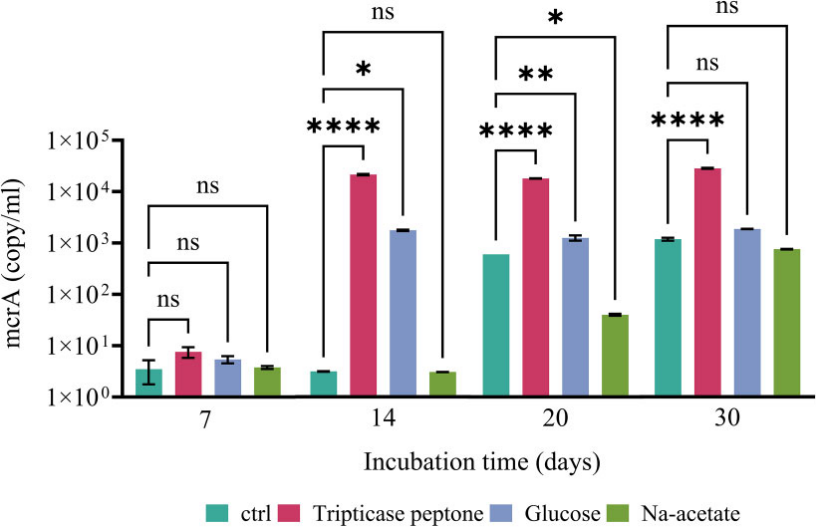
*mcrA* gene copy/ml with different carbon sources during the incubation time. The error bars show the standard deviation (*n* = 3). Statistical significance (*P*-value < .05) between treated and ctrl conditions is shown in the figure, while *P*-values are displayed in Table S5.

qPCR analyses were carried out on *mcrA, fhs*, and *dsrB* genes on the initial inoculum and at day 7, 14, 20, and 30 (Table S4). The initial inoculum showed an equal level of methanogenic archaea and acetogenic bacteria (*mcrA* 3.14 × 10^2^, *fhs* 3.71 × 10^2^ copy/ml). Conversely, in agreement with the hydro-chemical analyses, pointing out the low concentration of sulfates in formation water, *dsrB* gene was found below the detection limit, suggesting a negligible presence of SRB. As shown in Fig. 3 and Table S4 after 7 days incubation the number of copies of all investigated genes decreased. However, at day 14, the *mcrA* copy number significantly increased with trypticase peptone and glucose (*P*-value < .05) while the *fhs* copies dropped under the cutoff value. For the rest of the incubation period, the culture supplemented with trypticase peptone maintained the highest amount of *mcrA*, with a magnitude of 10^4^. On day 20, a significant (*P*-value < .05) rise in the copies number of *mcrA* to various degrees was detected for all conditions. At the end of the incubation time, only the trypticase condition showed a significant increase in *mcrA* copies compared to the control condition.

### Characterization of the most CH_4_ productive condition

Based on previous data, the condition supplemented with trypticase peptone appeared to be the most enriched in methanogenic archaea and provided the maximum yield of CH_4_. For these reasons, the original enriched culture was cultivated until day 60, and its evolution was studied using the same molecular biology and analytical methods as in the enrichment phase. This included pressure and gas measurements, scVFAs analysis, qPCR, amplicon sequencing of 16S rRNA V3–V4 region, and functional prediction using KEGG and COG analysis.

#### Microbial community structure

**Figure 4.**
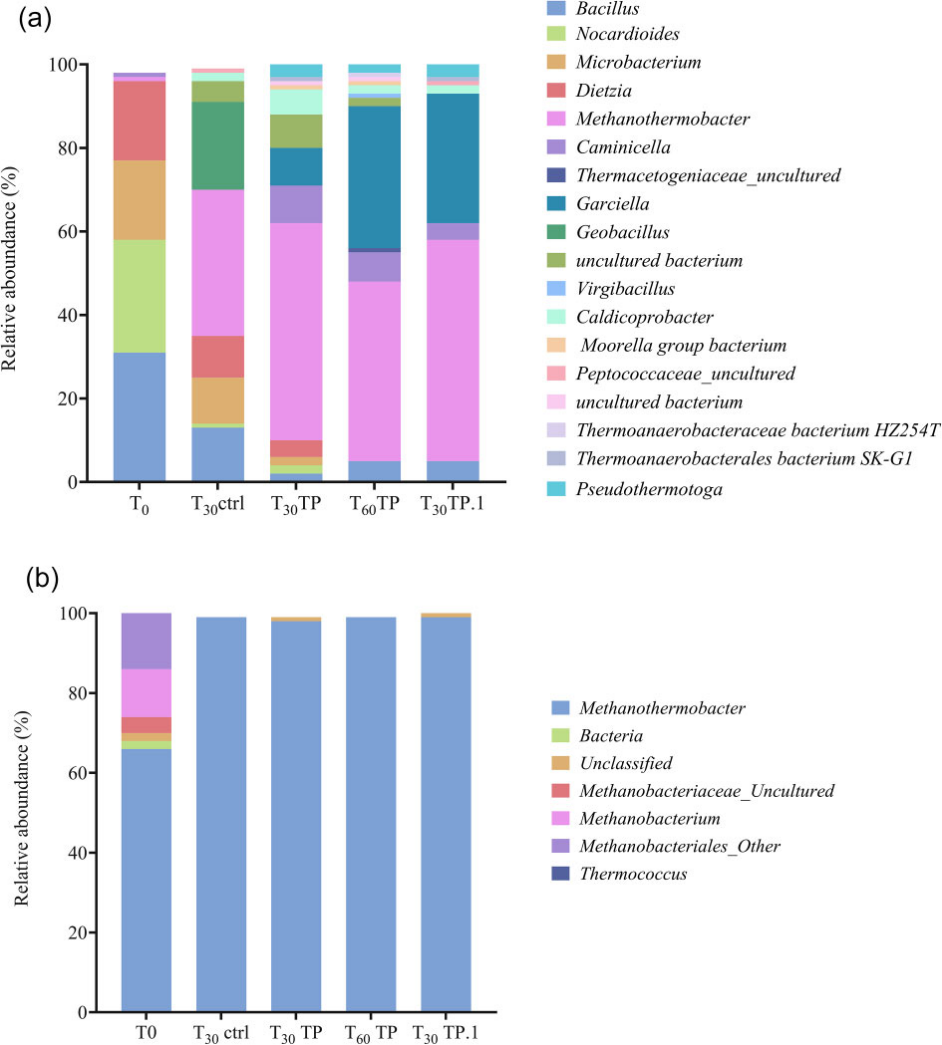
Overview of community structure at genus level. The community structure was derived from the 16S rRNA gene using bacterial (a) and archaeal (b) primers. Only taxa with an average relative abundance of ≥1% are displayed. Formation waters used as inoculum (T_0_), control (T_30_ctrl), and trypticase peptone conditions (T_30_TP) at the end of the incubation time, as well as trypticase peptone condition samples from the next stage of culture (T_60_TP and T_30_TP.1), were examined.

Microbial community analysis based on the sequencing of V3–V4 regions of 16S rRNA bacterial and archaeal genes, commonly used for microbial characterization of deep subsurface environmental samples (Cottier et al. 2018, Ghosh et al. 2019, Bassani et al. 2023), was performed to characterize the microbial community populating the formation water used as inoculum (T_0_), the batch culture control condition (T_30_ctrl) and the one supplemented with trypticase peptone at a different time during the experiment (T_30_TP, T_60_TP, T_30_TP.1) (Fig. 4). Detailed sample ID and sequencing results are listed in Table S6. Overall, 33 379 934 and 33 399 774 reads were produced for the bacterial and archaeal targets, respectively, with 70% and 76% of them being assigned to OTUs. The experimental conditions and the provision of H_2_/CO_2_ and trypticase peptone had a selective effect on the population, pushing towards a less diverse and possibly more specialized microbial community. This was demonstrated by both rarefaction curves and Shannon alpha diversity indexes (Fig. S1a and b), which indicated that the control and trypticase peptone conditions differed from each other and from those of the initial formation water sample. The beta diversity of the bacteria demonstrated how the control and trypticase peptone-added conditions produced consortia clustering separated from each other and especially from time zero (Fig. S1c and d). Conversely, the control and the peptone treatment for the target archaea clustered together, showing similarities within the archaea community, which, however, differed from the zero-time sample.

By looking at the most abundant taxa populating the formation water used as inoculum (T_0_), the microbial community was dominated by taxa classified as similar to *Bacillus* (36%), *Nocardoides* (26%), *Diezia* (18%), and *Microbacterium* (18%) genera. Interestingly, even though the analysis was carried out on a 16S rRNA bacterial target, a small percentage (1.37%) of the reads obtained with this target were assigned to archaeal members of the *Methanothermobacter* genus. Consistently, about 75% of the reads obtained from the analysis of 16S rRNA archaeal genes were assigned to the *Methanothermobacter* genus. After an incubation period of 30 days, the treatment with carbon sources led to a remarkable increment of the methanogenic population, and specifically the *Methanothermobacter* genus (Taxonomic assignments at genus level of 16S rRNA bacterial archaea gene are reported in Fig. 4 and Datasets 1–2). *Methanothermobacter* accounted for 34% and 50% of the total microbial community in the control and treated samples, respectively, analyzed with 16S rRNA bacterial target, and for about 100% of the community with the archaeal target. Additionally, the culture supplemented with trypticase peptone revealed notable percentages of *Pseudothermotoga* (3%), *Caldicoprobacter* (5.63%), *Caminicella*, and *Garciella* genera. Except for the genus Bacillus, all other species that dominated the initial sample were drastically reduced. The following culture steps with trypticase peptone, day 60 (T_60_TP) and the subculture at day 30 (T_30_TP.1), were dominated by *Methanothermobacterium* spp. (50%) and *Garciella* spp. (30%). *Caminicella* and *Pseudothermotogagenus* remained stable, while *Caldicoprotobacter* declined.

#### Predictive functional analysis of metabolic pathways in dominant genera

**Figure 5.**
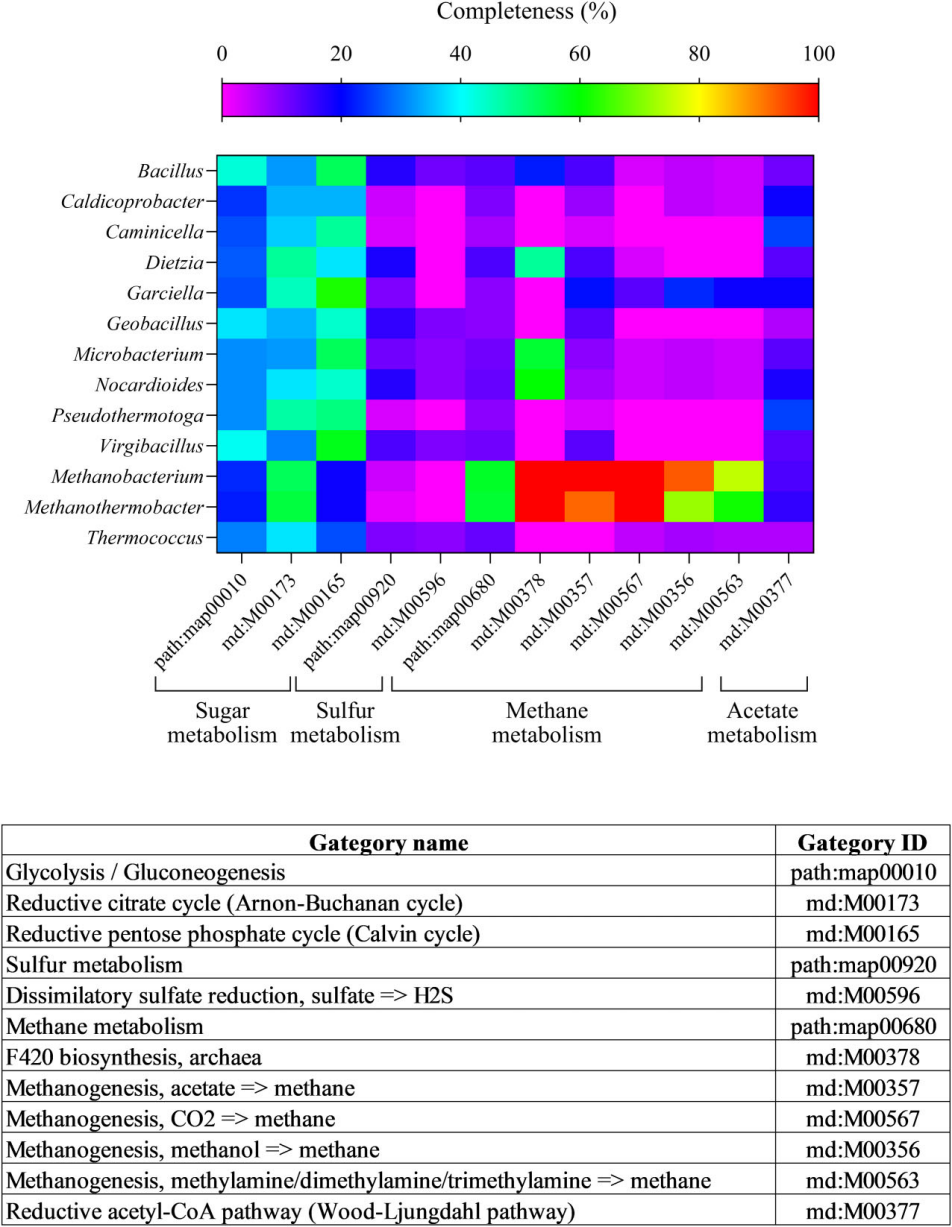
The heatmap illustrates the results of KEGG predictive metagenomic analysis. This analysis was conducted on the most prevalent bacterial and archaeal genera (relative abundance ≥1%) identified through 16S rRNA sequencing of batch culture treated with trypticase peptone. The heatmap reported the completeness of each KEGG category, calculated from the average number of genes assigned to each KEGG pathway or module per considered genome. The graph shows a selection of significant categories relevant to the description of syntrophic interaction in enriched cultures. Comprehensive KEGG and COG analysis is available in the Supplementary materials (Dataset 3)

Predictive functional analyses based on KEGG and COG categories (complete KEGG and COG analysis results are reported in the Dataset 3) were conducted to highlight metabolic pathways of potential interest in this study. This analysis for the trypticase peptone condition (Fig. 5) revealed a higher number of genes associated with hydrogenotrophic methanogenesis (∼43 genes; module ID: M00567) compared to those associated with other methanogenic routes (up to 31 and 16 genes for acetoclastic and methylotrophic pathways, respectively), confirming the hypothesis of the prevalence of this methanogenic metabolism in the culture, and thus HM. Furthermore, the analysis of the archaeal taxa revealed the presence of the whole pathway of cofactor F420 biosynthesis (module ID: M00378) which catalyzes important redox reactions in methanogenesis (Ferry 2011). As previously stated, the enriched culture consisted also of microorganisms classified as similar to *Garciella* (30%) and *Caminicella* (10%) genera, for which KEGG analysis revealed the nearly complete set of genes for the Wood–Ljundahl pathway (WLP; module ID: M00377) and the Reductive pentose phosphate cycle (Calvin cycle; module ID: M00165) which could justify the increase of VFA level during the incubation period.

#### Process performance and qPCR analysis

**Figure 6.**
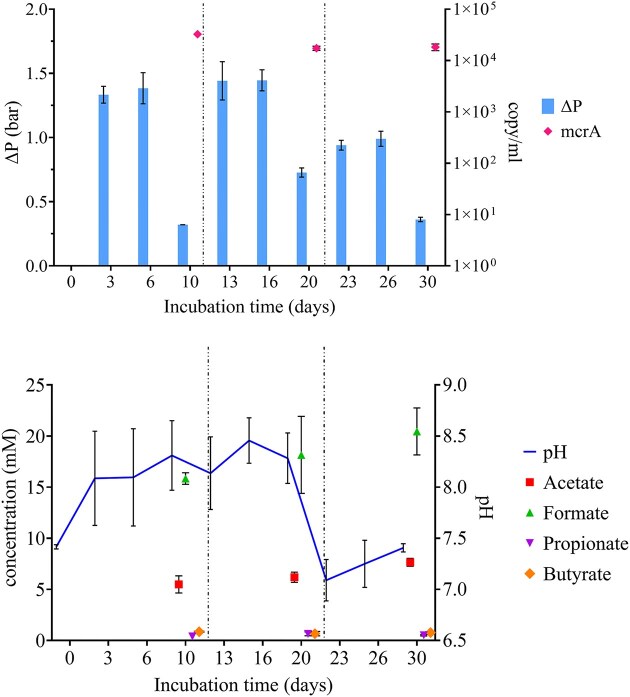
The panel illustrates the performance of the novel enriched culture, during further stages of cultivation. Gas consumption (∆*P*) was determined from the partial pressure difference between two successive measures, *mcrA* copy/ml (a); variation of VFAs concentration (mM) and pH during the incubation period of the first subculture (b). The dotted lines represent the refill with fresh mineral media. The error bars show the standard deviation (*n* = 3).

Culture headspace gas pressure measurements revealed a consistent pattern in gas consumption (Fig. 6a): methane production and consumption decreased after 10 days of incubation, then resumed following refilling with the fresh mineral medium. However, it can be noticed that consumption had substantially decreased during the last 10 days of incubation compared to the initial stages. This subculture's CH_4_ production rose by almost 39% compared to the initial culture, reaching up to 0.31 g CH_4_/g CO_2_. The *mcrA* copy/ml, and thus the amount of methanogens, maintained the same order of magnitude throughout the incubation period.

During the same period, variations in pH and concentration of VFAs were also evaluated (Fig. 6b). As observed during the screening experiment, the concentrations of formate and acetate were higher than propionate and butyrate. Formate concentrations ranged from 15.85 to 20.45 mM, while acetate from 5.50 to 7.65 mM. These concentrations seemed to be consistent with the first culture. The pH variation, which ranged from 7 to 8.5, remained in the optimal range for biomethanation. pH would seem to be influenced not only by fresh media refill but also by changes in gas pressure and composition in the headspace.

## Discussion

### Screening of batch cultures with different carbon sources

In all tested conditions, gas consumption was observed after a lag phase resulting from adaptation to the changed conditions. This lag phase reflects the time required to reduce the culture medium to a sufficiently low redox state in the absence of additional reducing agents, such as sodium sulfide (Na_2_S) or HCl-cysteine (Szafranek-Nakonieczna et al. 2019). In the present work, gas consumption and CH_4_ production appeared to be correlated with the relative abundance of the *mcrA* gene, as supported by the literature (Wang et al. 2018). Indeed, in environments in which sulfate levels are <50 µM, as the formation water and the mineral medium utilized in this study, syntrophic interactions between methanogens and fermentative microorganisms are favored over sulfate reducers and this is consistent with previous studies of the interactions of these microbial groups described in the literature (Oude Elferink et al. 1998). This dynamic relationship facilitates the efficient breakdown of organic matter and the production of CH_4_. Moreover, we showed how the type of carbon source provided has a significant impact on the enrichment strategy since it occurs according to the metabolic pathways of an anaerobic community. Results, reported here, suggested that CH_4_ production by microbial communities was significantly influenced by various organic carbon sources, with trypticase peptone being the most conducive, followed by glucose, acetate, and minimal production in the absence of added organic carbon. Trypticase peptone has been documented as a favorable carbon source for stimulating methanogenesis and it can significantly reduce the lag phase of methanogenesis, allowing for faster initiation of methane production compared to other carbon sources. Additionally, it has been observed to support higher maximum methane production rates, indicating its potential for enhancing overall methanogenic activity (Xing et al. 2017). The reason behind the effectiveness of trypticase peptone lies in its composition, which provides a readily available source of nutrients for methanogenic microorganisms. According to the literature, the addition of acetate induce less methane and much later (Luo et al. 2022). Glucose itself does not produce CH_4_ but can be absorbed and converted into various scVFA through microbial fermentation. scVFA, such as acetate, propionate, and butyrate, are key intermediates in anaerobic fermentation and have different CH_4_ production capabilities. The conversion of organic matter typically necessitates a microbial consortium consisting of three or more species of microorganisms. Studying the dynamics of scVFA production enables us to understand the impact of various organic sources on the fermentation process and to elucidate the syntrophic relationships that can develop within microbial consortia. The observed increase in acetate concentration, coupled with low concentrations of other fatty acids, could indeed indicate the presence of syntrophic acetate oxidizers (SAOs) and syntrophic fatty acid oxidizers (scFAOs) (Saha et al. 2020, Ferry 2011). These microorganisms are specialized in oxidizing complex fatty acids to acetate, thereby contributing to acetate production within the microbial consortium. In this study, the low concentrations of propionate and butyrate suggested that these microorganisms might convert them into acetate and/or formate. However, the lack of acetate consumption suggests the absence of acetoclastic methanogens, which typically consume acetate as a substrate for CH_4_ production. Instead, the activity of fermentative microorganisms is supported by the presence of HM which maintain the H_2_ partial pressure below 10^2^ Pa (Nazaries et al. 2013, Saha et al. 2020). The lower H_2_ partial pressure, as measured by headspace pressures, is thought to stimulate exergonic reactions of acetogenic microorganisms capable of converting butyrate to acetate and propionate to acetate or formate (Shen et al. 2016, Saha et al. 2020). This relationship between HM and acetogenic microorganisms would account for the low concentrations of butyrate and propionate, as well as the increase in formate and acetate concentrations at day 14 when a significant pressure drop was detected in the conditions supplemented with trypticase peptone and glucose.

### Characterization of the most CH_4_ productive condition

The results of the community analysis revealed a reduction in diversity during the incubation period compared to the inoculum sample, with a remarkable increase of reads assigned to the *Methanothermobacter* genus in all analyzed conditions. This trend is consistent with the experimental enrichment approach we used. Indeed, the observed increase in *Methanothermobacte*r abundance could be attributed to the effect of a gas mix rich in H_2_, which promotes the enrichment of HM (Bassani et al. 2015, 2017). These methanogens utilize H_2_ as a key substrate for CH_4_ production. In accordance, KEGG analysis predicted the presence of a higher number of genes associated with this metabolic pathway, compared to the other methanogenic routes. Compared with the control, the enriched culture supplemented with trypticase peptone revealed a different consortium of bacteria resembling *Pseudothermotoga, Caldicoprotobacter, Camininicella*, and *Garciella* genera, as well as a greater percentage of reads attributable to *Methanotherobacter* genus. Genus selection can be ascribed not only to the carbon supply (i.e. trypticase peptone), but also to other parameters such as neutral pH and temperature that are known to promote the growth of the aforementioned genera of fermenting microorganisms. KEGG and COG analyses predicted a nearly complete set of genes for WLP and Calvin Cycle for genera *Garciella* and *Caminicella*, which are heterotrophic bacteria unable to grow on homoacetogenic pathways. According to the literature, they can ferment complex carbohydrate and proteinaceous substrates such as the trypticase peptone, producing H_2_ and CO_2_ but also acetate and formate and other scVFA (Alain et al. 2002, Miranda-Tello et al. 2003). These findings supported the data obtained from the subculture characterization and led us to hypothesize a syntrophic relationship between *Metanothermobacter, Garciella*, and *Caminicella* spp. In a syntrophic scenario, H_2_ partial pressure could be kept below 10^2^ Pa by HM, allowing acetogens to react in an exergonic reaction that converts propionate and butyrate into acetate or formate (Bellini et al. 2024). This hypothesis could be supported by the fact that acetate and formate synthesis began in correlation with the decline in pressure on day 14 of incubation and remained constant throughout the incubation time and for the subculture. The absence of acetoclastic methanogens could be a simple explanation for acetate accumulation. During the interspecies electron transfer process, formate and H_2_ operate as electron carriers; interspecies hydrogen transfer (IHT) and interspecies formate transfer (IFT) can occur simultaneously. Literature suggests that formate serves as a crucial diffusive redox mediator in methanogenic communities, particularly due to its higher solubility (Hattori et al. 2001, Shen et al. 2016, Saha et al. 2020). IFT tends to dominate when interspecies distances are significant. Conversely, when distances between species are minimal, IHT becomes more favorable. This leads us to speculate that the accumulation of formate resulted from a preferential IHT, facilitated by the formation of methanogenic aggregates which represent functional units comprising diverse microorganisms essential for the methanogenic degradation of organic matter (Shen et al. 2016).

## Conclusions

This study explored a new enrichment strategy and diverse carbon sources for stimulating methanogenesis using formation water sampled from an underground gas storage in the perspective of using depleted reservoirs as biomethanation reactors. The first outcome of this study is that the supply of trypticase peptone facilitated significantly methane production, surpassing other carbon sources. Gas consumption and CH_4_ production are correlated to the abundance of the *mcrA* gene, highlighting how the carbon source type influences the methane production yield. The microbial characterization of the enriched consortium obtained when trypticase peptone was supplied showed shifts in the microbial community structure, with the *Methanothermobacter* dominating alongside other genera while the functional pathway analysis indicated the prevalence of hydrogenotrophic methanogenesis, consistent with our experimental data. Syntrophic interactions between *Methanothermobacter* spp. and fermentative bacteria belonging to the *Garciella* and *Caminicella* genus were assumed, facilitating efficient degradation of the organic matter. The investigated reservoir emerged as a promising site for UMR implementation with its favorable hydrochemical properties and high cell number of methanogenic archaea. This work provides the basis for biologically exploiting the selected depleted gas reservoir for UMR technology, focusing on the by activation of indigenous methanogenic archaea or bioaugmentation strategy. Indeed, the CH_4_ yield and self-reduction capacity observed in the enriched consortium indicated its potential; however, further investigation under pressure conditions close to those of deep geological formations are needed to prove the UMR feasibility. Further experimental investigations will be carried out in a reactor test to evaluate the enriched methanogenic culture at a higher pressure because the H_2_ consumption for microbial growth in serum flask assays at 2 bar is limited by H_2_ low dissolution. It will also be necessary to decrease trypticase peptone concentration and adapt the culture at this higher pressure. Media optimization is also required because the complex composition of trypticase may overstimulate the growth of undesirable microorganisms and subsequently cause biochemical issues, such as pore clogging (Eddaoui et al. 2021).

## Consent for publication

All the authors avail and consent the publication of the present work.

## Supplementary Material

fiaf040_Supplemental_Files

## Data Availability

The data for this study have been deposited in the European Nucloeotide Archive (ENA) at EMBL-EBI under accession number PRJEB76268.
