# Supplementary material for: Assessing the methanogenic activity of microbial communities enriched from a depleted reservoir
Source: FEMS Microbiol Ecol. 2025 Apr 15;101(5):fiaf040. doi: 10.1093/femsec/fiaf040 (PMC12054477; doi:10.1093/femsec/fiaf040)
Supplement: fiaf040_Supplemental_Files [file fiaf040_supplemental_files.zip › SUPPLEMENTARY_MATERIAL_revisedV2.docx]

***Assessing the methanogenic activity of microbial communities enriched from a depleted reservoir***

Arianna Vizzarro ^1,2^, Annalisa Abdel Azim^3^, Ilaria Bassani^1^, Ruggero Bellini^1^, Nicolò Santi Vasile^2^, Candido Fabrizio Pirri,^1,3^ Francesca Verga^2^, Barbara Menin^1,4^

^1^ Centre for Sustainable Future Technologies, Fondazione Istituto Italiano di Tecnologia, Via Livorno 60, 10144 Turin, Italy.

^2^ Politecnico di Torino, Department of Environment, Land and Infrastructure Engineering, Corso Duca degli Abruzzi 24, 10129 Turin, Italy

^3^ Politecnico di Torino^,^ Department of Applied Science and Technology, Corso Duca degli Abruzzi 24, 10129 Turin, Italy.

^4^ Istituto di Biologia e Biotecnologia Agraria, Consiglio Nazionale delle Ricerche, Via Alfonso Corti 12, 20133 Milan, Italy.

*** Correspondence:** Ruggero Bellini
 ruggero.bellini@iit.it

This supplementary material includes:

Table S1-S5

References

**Table S1**: Idrochemical analysis

| **Parameters (mg/l)** | **sample 1** | **sample 2** | **sample 3** | **Avarage** | **St.dev** | **SEM** | **Method** |
| --- | --- | --- | --- | --- | --- | --- | --- |
| **NH4** | 49 | 48 | 49 | 48.67 | 0.58 | 0.33 | UNI 11669:2017 |
| **PO4** | <8 | <8 | <8 | <8 | 0.00 | 0.00 | APAT CNR IRSA 2060 Man29 2003 |
| **Chloride** | 20061 | 18978 | 19056 | 19365.00 | 604.01 | 348.73 | APAT CNR IRSA 2060 Man29 2004 |
| **Sulphate** | <50 | <50 | <50 | <50 | 0.00 | 0.00 | APAT CNR IRSA 2060 Man29 2005 |
| **Nitrate** | <50 | <50 | <50 | <51 | 0.00 | 0.00 | APAT CNR IRSA 2060 Man29 2006 |
| **Bromide** | 100 | 100 | 109 | 103.00 | 5.20 | 3.00 | APAT CNR IRSA 2060 Man29 2007 |
| **Bicarbonate** | 131.04 | 124.32 | 127.68 | 127.68 | 3.36 | 1.94 | APAT CNR IRSA 2060 Man29 2008 |
| **Total organic carbon** | 10.36 | 12.48 | 20.56 | 14.47 | 5.38 | 3.11 | UNI EN 1484:1999 |
| **Total inorganic carbon** | 80.24 | 79.68 | 60.68 | 73.53 | 11.13 | 6.43 | UNI EN 1484:2000 |
| **Total carbon** | 90.6 | 92.16 | 81.24 | 88.00 | 5.91 | 3.41 | UNI EN 1484:2001 |
| **Calcium** | 510 | 500 | 510 | 506.67 | 5.77 | 3.33 | UNI EN ISO 11885:2009 |
| **Magnesium** | 313 | 304 | 309 | 308.67 | 4.51 | 2.60 | UNI EN ISO 11885:2010 |
| **Sodium** | 9600 | 9300 | 9500 | 9466.67 | 152.75 | 88.19 | UNI EN ISO 11885:2011 |
| **Potassium** | 161 | 156 | 156 | 157.67 | 2.89 | 1.67 | UNI EN ISO 11885:2012 |
| **Lithium** | 2.341 | 2.283 | 2.388 | 2.34 | 0.05 | 0.03 | UNI EN ISO 11885:2013 |
| **Strontium** | 18.468 | 17.904 | 18.708 | 18.36 | 0.41 | 0.24 | UNI EN ISO 11885:2014 |
| **Manganese** | 0.399 | 0.824 | 0.551 | 0.59 | 0.22 | 0.12 | UNI EN ISO 11885:2015 |
| **Nickel** | 0.007 | 0.009 | 0.007 | 0.01 | 0.00 | 0.00 | UNI EN ISO 11885:2016 |
| **Iron** | <5 | <5 | <5 | <5 | 0.00 | 0.00 | UNI EN ISO 11885:2017 |
| **pH** | 7.33 | 7.38 | 7.34 | 7.35 | 0.03 | 0.02 | APAT CNR IRSA 2060 Man29 2003 |

**Table S2**: List of primer sequences used in this study.

| **Primer name** | **Oligonucleotide sequence (5’–3’)** | **Reference** |
| --- | --- | --- |
| **357wF** | CCTACGGGNGGCWGCAG | Herlemann *et al.*, 2011 |
| **785R** | GACTACHVGGGTATCTAATCC | Klindworth *et al.*, 2013 |
| **Arch340wF** | CCCTAYGGGGYGCASCAG | Gantner *et al.*, 2011 |
| **Arch806R** | GGACTACVSGGGTATCTAAT | Takai and Horikoshi, 2000 |
| **DSRp2060F** | CAACATCGTYCAYACCCAGGG | Foti *et al.*, 2007 |
| **DSR4R** | GTGTAGCAGTTACCGCA | Foti *et al.*, 2007 |
| **ME1** | GCMATGCARATHGGWATGTC | Hales *et al.*, 1996 |
| **ME3R** | TGTGTGAAWCCKACDCCACC | Nyyssönen *et al.*, 2012 |
| **fhs2** | GARGAYGGWTTTGAYATYAC | Xu *et al.*, 2009 |
| **FTHFS-r** | GTATTGDGTYTTRGCCATACA | Leaphart *et al.,* 2001 |

**Table S3**: Statistical significance (p-value < 0,05) of gas consumption between different experimental conditions and the control condition, assessed using one-way ANOVA, Tukey's multiple comparisons test, with a single

|  |  | **Tukey's multiple comparisons test** | **Adjusted P Value** | **Summary** |
| --- | --- | --- | --- | --- |
| Incubation time (days) | 7 | ctrl vs. T-peptone | 0.8857 | ns |
|  |  | ctrl vs. Glucose | 0.9252 | ns |
|  |  | ctrl vs. Na- Acetate | 0.0299 | * |
|  |  | T-peptone vs. Glucose | 0.8583 | ns |
|  |  | T-peptone vs. Na- Acetate | 0.9293 | ns |
|  |  | Glucose vs. Na- Acetate | 0.6194 | ns |
|  | 14 | ctrl vs. T-peptone | 0.0243 | * |
|  |  | ctrl vs. Glucose | 0.5324 | ns |
|  |  | ctrl vs. Na- Acetate | 0.0924 | ns |
|  |  | T-peptone vs. Glucose | 0.1551 | ns |
|  |  | T-peptone vs. Na- Acetate | 0.0242 | * |
|  |  | Glucose vs. Na- Acetate | 0.5412 | ns |
|  | 20 | ctrl vs. T-peptone | 0.0137 | * |
|  |  | ctrl vs. Glucose | 0.7088 | ns |
|  |  | ctrl vs. Na- Acetate | 0.7683 | ns |
|  |  | T-peptone vs. Glucose | 0.0924 | ns |
|  |  | T-peptone vs. Na- Acetate | 0.0795 | ns |
|  |  | Glucose vs. Na- Acetate | 0.4451 | ns |
|  | 30 | ctrl vs. T-peptone | 0.0199 | * |
|  |  | ctrl vs. Glucose | 0.9276 | ns |
|  |  | ctrl vs. Na- Acetate | 0.9645 | ns |
|  |  | T-peptone vs. Glucose | 0.1695 | ns |
|  |  | T-peptone vs. Na- Acetate | 0.0144 | * |
|  |  | Glucose vs. Na- Acetate | 0.9935 | ns |

**Table S4:** Normalized avarage of mcrA, fhs and dsrb copies/ml with different carbon sources. . The qPCR method yielded the lowest amplicon concentrations: 1.33 x 102, 1.85 x 102, and 1.76 x 102 copies/mL for mcrA, dsrB, and fhs, respectively. As a result, samples exhibiting quantities below these cutoff points are labelled as “not determined (n.d.).”

|  |  | **Conditions** | **mcrA (copies/ml)** | **fhs (copies/ml)** | **dsrb (copies/ml)** |
| --- | --- | --- | --- | --- | --- |
| **Incubation time (days)** | 0 | Inoculum | 3,14E+02 | 3,71E+02 | n.d. |
|  | 7 | Control | 3,49E+00 | 1,09E+00 | n.d. |
|  |  | Trypticase peptone | 7,58E+00 | 5,92E+00 | n.d. |
|  |  | Glucose | 5,41E+00 | 7,64E+00 | n.d. |
|  |  | Na- acetate | 3,77E+00 | 5,92E+00 | n.d. |
|  | 14 | Control | 1,11E+00 | n.d. | n.d. |
|  |  | Trypticase peptone | 2,03E+04 | 1,04E+01 | n.d. |
|  |  | Glucose | 1,69E+03 | n.d. | n.d. |
|  |  | Na- acetate | 2,75E-02 | n.d. | n.d. |
|  | 20 | Control | 5,79E+02 | 8,25E+00 | n.d. |
|  |  | Trypticase peptone | 1,71E+04 | 1,20E+01 | n.d. |
|  |  | Glucose | 1,21E+03 | 3,60E+00 | n.d. |
|  |  | Na- acetate | 3,92E+01 | n.d. | n.d. |
|  | 30 | Control | 1,14E+03 | 1,49E+01 | n.d. |
|  |  | Trypticase peptone | 2,70E+04 | 6,44E+01 | n.d. |
|  |  | Glucose | 1,80E+03 | n.d. | n.d. |
|  |  | Na- acetate | 7,33E+02 | n.d. | n.d. |

**Table S5**: Statistical significance (p-value < 0,05) of mcrA copies/ml between different experimental conditions and the control condition, assessed using one-way ANOVA, Tukey's multiple comparisons test, with a single pooled

|  |  | **Tukey's multiple comparisons test** | **P Value** | **Summary** |
| --- | --- | --- | --- | --- |
| Incubation time (days) | 7 | ctrl vs. Tripticase peptone | 0.2002 | ns |
|  |  | ctrl vs. Glucose | 0.7339 | ns |
|  |  | ctrl vs. Na-acetate | 0.9987 | ns |
|  |  | Tripticase peptone vs. Glucose | 0.6592 | ns |
|  |  | Tripticase peptone vs. Na-acetate | 0.2448 | ns |
|  |  | Glucose vs. Na-acetate | 0.8123 | ns |
|  | 14 | ctrl vs. Tripticase peptone | <0,0001 | **** |
|  |  | ctrl vs. Glucose | 0.0236 | * |
|  |  | ctrl vs. Na-acetate | >0,9999 | ns |
|  |  | Tripticase peptone vs. Glucose | <0,0001 | **** |
|  |  | Tripticase peptone vs. Na-acetate | <0,0001 | **** |
|  |  | Glucose vs. Na-acetate | 0.0236 | * |
|  | 20 | ctrl vs. Tripticase peptone | <0,0001 | **** |
|  |  | ctrl vs. Glucose | 0.0066 | ** |
|  |  | ctrl vs. Na-acetate | 0.0159 | * |
|  |  | Tripticase peptone vs. Glucose | <0,0001 | **** |
|  |  | Tripticase peptone vs. Na-acetate | <0,0001 | **** |
|  |  | Glucose vs. Na-acetate | 0.0001 | *** |
|  | 30 | ctrl vs. Tripticase peptone | <0,0001 | **** |
|  |  | ctrl vs. Glucose | 0.4586 | ns |
|  |  | ctrl vs. Na-acetate | 0.7796 | ns |
|  |  | Tripticase peptone vs. Glucose | <0,0001 | **** |
|  |  | Tripticase peptone vs. Na-acetate | <0,0001 | **** |
|  |  | Glucose vs. Na-acetate | 0.1357 | ns |

**Table S6**: List of samples analyzed by 16S rRNA bacterial and archaeal gene sequencing and summary of sequencing results obtained for each biological and technical replicate.

|  |  |  | 16S rRNA bacterial gene | | | | 16S rRNA archaeal gene | | | |
| --- | --- | --- | --- | --- | --- | --- | --- | --- | --- | --- |
| Sample N° | Sample ID | Sample Source | BioSample ID | Reads sequenced | Reads assigned to OTUs | Reads assigned to OTUs (%) | BioSample ID | Reads sequenced | Reads assigned to OTUs | Reads assigned to OTUs (%) |
| **1** | T0-a | Initial inoculum | SAMEA115672667 | 2308208 | 1678986 | 73% | SAMEA115672674 | 553916 | 441666 | 80% |
| **2** | T0-b |  |  | 1690676 | 1211110 | 72% |  | 828416 | 612596 | 74% |
| **3** | T0-c |  |  | 1400074 | 921292 | 66% |  | 1943008 | 1511092 | 78% |
| **4** | T30-ctrl-a | Control culture at T30 | SAMEA115672668 | 1307380 | 1008574 | 77% | SAMEA115672674 | 1068768 | 933494 | 87% |
| **5** | T30-ctrl-b |  |  | 1467252 | 1152592 | 79% |  | 1762856 | 1503494 | 85% |
| **6** | T30-ctrl-c |  |  | 1537584 | 1172800 | 76% |  | 650874 | 560264 | 86% |
| **7** | T30-TP-a | Tripticase culture at T30 | SAMEA115672669 | 1613728 | 1137064 | 70% | SAMEA115672675 | 1579684 | 1407650 | 89% |
| **8** | T30-TP-b |  |  | 1351152 | 934092 | 69% |  | 1607680 | 1413228 | 88% |
| **9** | T30-TP-c |  |  | 1335070 | 985060 | 74% |  | 1780482 | 1554860 | 87% |
| **10** | T60-TP.A-a | Tripticase culture A at T60 | SAMEA115672670 | 724210 | 512240 | 71% | SAMEA115672676 | 1318584 | 1149528 | 87% |
| **11** | T60-TP.A-b |  |  | 1364070 | 920534 | 67% |  | 2515676 | 2232484 | 89% |
| **12** | T60-TP.A-c |  |  | 1503194 | 1025608 | 68% |  | 877554 | 777442 | 89% |
| **13** | T60-TP.B-a | Tripticase culture B at T60 | SAMEA115674993 | 1836024 | 1242336 | 68% | SAMEA115674995 | 1279960 | 1128416 | 88% |
| **14** | T60-TP.B-b |  |  | 2011956 | 1378114 | 68% |  | 1417474 | 1238884 | 87% |
| **15** | T60-TP.B-c |  |  | 1681418 | 1162398 | 69% |  | 1091196 | 964352 | 88% |
| **16** | T30-TP.1.A-a | Tripticase subculture A at T30 | SAMEA115672671 | 1439548 | 998592 | 69% | SAMEA115672677 | 1019890 | 902656 | 89% |
| **17** | T30-TP.1.A-b |  |  | 746532 | 535194 | 72% |  | 969094 | 820142 | 85% |
| **18** | T30-TP.1.A-c |  |  | 1935750 | 1354868 | 70% |  | 723156 | 643246 | 89% |
| **19** | T30-TP.1.B-a | Tripticase subculture B at T30 | SAMEA115674994 | 1530074 | 1077972 | 70% | SAMEA115674996 | 925984 | 811988 | 88% |
| **20** | T30-TP.1.B-b |  |  | 1416492 | 1030674 | 73% |  | 769258 | 681148 | 89% |
| **21** | T30-TP.1.B-c |  |  | 1239722 | 806754 | 65% |  | 1831800 | 1595276 | 87% |
| **22** | lab-ctrl-a | LAB CONTROL (elution buffer used as substrate) | SAMEA115672672 | 712610 | 453132 | 64% | SAMEA115672678 | 3853328 | 140652 | 4% |
| **23** | lab-ctrl-b |  |  | 1011198 | 669752 | 66% |  | 2408436 | 87534 | 4% |
| **24** | lab-ctrl-c |  |  | 216012 | 124242 | 58% |  | 622700 | 74106 | 12% |

**Fig. S1** Rarefaction curves of annotated OTUs diversity and beta diversity PCoA plots. They were generated from 16S rRNA bacterial (a and c) and archaeal (b and d) gene sequencing analyses. Detailed sample ID and sequencing results are listed in Table S6.


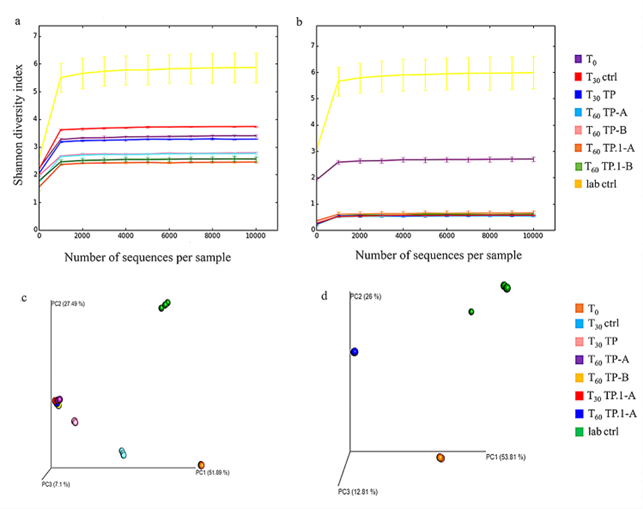


**References**

Foti, M., Sorokin, D. Y., Lomans, B., Mussman, M., Zacharova, E. E., Pimenov, N. V., Kuenen, J. G., & Muyzer, G. (2007). Diversity, activity, and abundance of sulfate-reducing bacteria in saline and hypersaline soda lakes. *Applied and Environmental Microbiology*, *73*(7), 2093–2100. https://doi.org/10.1128/AEM.02622-06

Foti et al., 2007; Gantner et al., 2011; Hales et al., 1996; Herlemann et al., 2011; Klindworth et al., 2013; Nyyssönen et al., 2012; Takai & Horikoshi, 2000; Xu et al., 2009)

Gantner, S., Andersson, A. F., Alonso-Sáez, L., & Bertilsson, S. (2011). Novel primers for 16S rRNA-based archaeal community analyses in environmental samples. *Journal of Microbiological Methods*, *84*(1), 12–18. https://doi.org/https://doi.org/10.1016/j.mimet.2010.10.001

Hales, B. A., Edwards, C., Ritchie, D. A., Hall, G., Pickup, R. W., & Saunders, J. R. (1996). Isolation and identification of methanogen-specific DNA from blanket bog peat by PCR amplification and sequence analysis. *Applied and Environmental Microbiology*, *62*(2), 668–675. https://doi.org/10.1128/aem.62.2.668-675.1996

Herlemann, D. P. R., Labrenz, M., Jürgens, K., Bertilsson, S., Waniek, J. J., & Andersson, A. F. (2011). Transitions in bacterial communities along the 2000 km salinity gradient of the Baltic Sea. *ISME Journal*, *5*(10), 1571–1579. https://doi.org/10.1038/ismej.2011.41

Klindworth, A., Pruesse, E., Schweer, T., Peplies, J., Quast, C., Horn, M., & Glöckner, F. O. (2013). Evaluation of general 16S ribosomal RNA gene PCR primers for classical and next-generation sequencing-based diversity studies. *Nucleic Acids Research*, *41*(1), 1–11. https://doi.org/10.1093/nar/gks808

Leaphart, A. B., & Lovell, C. R. (2001). Recovery and Analysis of Formyltetrahydrofolate Synthetase Gene Sequences from Natural Populations of Acetogenic Bacteria. *Applied and Environmental Microbiology*, *67*(3), 1392–1395. https://doi.org/10.1128/AEM.67.3.1392-1395.2001

Nyyssönen, M., Bomberg, M., Kapanen, A., Nousiainen, A., Pitkänen, P., & Itävaara, M. (2012). Methanogenic and Sulphate-Reducing Microbial Communities in Deep Groundwater of Crystalline Rock Fractures in Olkiluoto, Finland. *Geomicrobiology Journal*, *29*(10), 863–878. https://doi.org/10.1080/01490451.2011.635759

Takai, K., & Horikoshi, K. (2000). Rapid detection and quantification of members of the archaeal community by quantitative PCR using fluorogenic probes. *Applied and Environmental Microbiology*, *66*(11), 5066–5072. https://doi.org/10.1128/AEM.66.11.5066-5072.2000

Xu, K., Liu, H., Du, G., & Chen, J. (2009). Real-time PCR assays targeting formyltetrahydrofolate synthetase gene to enumerate acetogens in natural and engineered environments. *Anaerobe*, *15*(5), 204–213. https://doi.org/https://doi.org/10.1016/j.anaerobe.2009.03.005
